# The effects of a 6-week controlled, hypocaloric ketogenic diet, with and without exogenous ketone salts, on cognitive performance and mood states in overweight and obese adults

**DOI:** 10.3389/fnins.2022.971144

**Published:** 2022-09-30

**Authors:** Madison L. Kackley, Milene L. Brownlow, Alex Buga, Chris D. Crabtree, Teryn N. Sapper, Annalouise O’Connor, Jeff S. Volek

**Affiliations:** ^1^Department of Human Sciences, The Ohio State University, Columbus, OH, United States; ^2^Research and Development Department, Metagenics Inc., Gig Harbor, WA, United States

**Keywords:** mood, cognition, ketogenic diet, BHB, ketone salts, keto-adaptation, BDNF, sodium

## Abstract

**Background:**

Ketogenic diets are a commonly used weight loss method, but little is known how variations in sodium content and ketones influence cognition and mood during the early keto-adaptation period.

**Objectives:**

To investigate the effects of an exogenous ketone salt (KS) as part of a hypocaloric KD on mood and cognitive outcomes in overweight and obese adults. A secondary objective was to evaluate changes in biochemical markers associated with inflammatory and cognitive responses.

**Materials and methods:**

Adults who were overweight or obese participated in a 6-week controlled-feeding intervention comparing hypocaloric diets (∼75% of energy expenditure). KD groups received twice daily ketone salt (KD + KS; *n* = 12) or a flavor-matched placebo, free of minerals (KD + PL; *n* = 13). A separate group of age and BMI matched adults were later assigned to an isoenergetic low-fat diet (LFD; *n* = 12) as comparison to KD. Mood was assessed by shortened Profile of Mood States and Visual Analog Mood Scale surveys. Cognitive function was determined by the Automated Neuropsychological Assessment Metrics mental test battery.

**Results:**

Both KD groups achieved nutritional ketosis. Fasting serum glucose decreased in both KD groups, whereas glucose was unaffected in the LFD. Insulin decreased at week 2 and remained lower in all groups. At week 2, depression scores in the KD + PL group were higher compared to KD + KS. Performance in the math processing and go/no-go cognitive tests were lower for KD + PL and LFD participants, respectively, compared to KD + KS. Serum leptin levels decreased for all groups throughout the study but were higher for KD + KS group at week 6. Serum TNF-α steadily increased for LFD participants, reaching significance at week 6.

**Conclusion:**

During a short-term hypocaloric diet, no indication of a consistent decline in mood or cognitive function were seen in participants following either KD, despite KD + PL being relatively low in sodium. WK2 scores of “anger” and “depression” were higher in the LFD and KD + PL groups, suggesting that KS may attenuate negative mood parameters during the early intervention stages.

## Introduction

Poor metabolic health contributes to impaired cognitive and behavioral performance. KD have shown consistent benefits to metabolic health and have been clinically studied in neurodegenerative diseases, such as epilepsy, Alzheimer’s disease (AD) and Parkinson’s disease, among others (Huttenlocher, 1976; VanItallie et al., 2005; Taylor et al., 2018). KDs have resulted in favorable outcomes in cognitive improvement post intervention (Croteau et al., 2018; Fortier et al., 2019). Recently, a KD was administered to adults who had been admitted to a psychiatric hospital and were suffering from various mental disorders. The dietary intervention lasted between 16 and 248 days and showed significant improvements in depression and psychotic symptoms (Danan et al., 2022). However, mood and cognitive side effects of the initial adaptation process associated with a KD have been less studied. Common symptoms at the onset of a KD include headache, dizziness, nausea, vomiting, fatigue, low exercise tolerance and constipation, colloquially referred to as the “keto flu.” One review found that out of 300 participants across an open forum, 101 participants reported the aforementioned symptoms of “keto-flu,” particularly in the first 4 weeks following the onset of the KD. Almost half of those reported flu-like symptoms; 24.8% reported headaches, and 17.8% suffered from fatigue (Bostock et al., 2017). Given the self-reported nature of their study, a major limitation is the lack of reporting of ketone levels and likelihood that many people were not following well-formulated KDs.

Flu-like symptoms on a KD may be primarily attributed to hypovolemia, secondary to inadequate sodium and fluid intake to compensate for the natriuretic and diuretic effect long known to occur in starvation ketosis (Stinebaugh and Schloeder, 1966) and the KD (Bloom and Azar, 1963). There are also robust metabolic adaptations occurring during the first several weeks of a KD that contribute to a marked decrease in glucose utilization and increased reliance on fatty acids and ketones for fuel. The “keto-adaptation” process that involves major changes in cellular fueling and altered mineral balances may predispose individuals consuming a KD to adverse effects on mood and/or cognition during the early phases of weight loss (Johnston et al., 2006). Negative effects of a KD during early phase adaptations could be minimized or eliminated by adhering to components of a well-formulated ketogenic diet (Volek et al., 2021); namely formulating the diet with attention to appropriate carbohydrate and protein intake from a variety of food sources, adequate sodium, and potassium to meet requirements, quality fat sources, and avoidance of intense exercise.

In this exploratory study we assessed serial measures of mood and cognition in healthy individuals starting a well-formulated KD for the first time. Here we evaluated whether supplementing with a ketone salt (KS) providing a direct dietary source of BHB and minerals (principally sodium) during the KD had any influence on mood and cognition during the early phase of weight loss. We included a matched group of individuals fed a low-fat diet with matched caloric restriction as a control comparison. To enhance compliance and ensure standardized nutrient intakes across groups and over time, we provided all food to participants over 6-weeks.

## Materials and methods

### Study design and human participants

The study participants, experimental diet interventions, and body composition results have been previously reported (Buga et al., 2021; Crabtree et al., 2021). In brief, this controlled prospective feeding study was a placebo-controlled, double-blind trial. The primary aim was to assess the effects of a KD with exogenous ketone salt supplementation (KD + KS, *n* = 12, 6 men/6 women) and a KD with a flavor matched placebo (KD + PL, *n* = 13, 6 men/7 women) on mood, cognition, and related biomarkers. A BMI and age matched group of participants was later assigned to an energy- and protein-matched low-fat diet (LFD, *n* = 12). Eligibility was determined based on age (21–65 years) and BMI (27–35 kg/m^2^). Exclusion criteria included: >10% weight loss in the 6-month period prior to enrollment, endocrine disfunction, drug use, smoking, alcoholism, epilepsy, headaches, pregnancy, usage of antibiotic/antifungal medication, or if currently on a ketogenic diet. Eligible participants completed a food frequency questionnaire, medical history questionnaire, and a physical activity questionnaire before they signed an informed consent document approved by the Ohio State University institutional review board (IRB #2017H0395). There were no significant differences between groups in baseline characteristics (Table 1).

**TABLE 1:** Participant characteristics.

	KD + KS (n-12)	KD + PL (*n* = 13)	LFD (*n* = 12)	*P*-value
Sex (male/female)	6/6	6/7	6/6	
Age Range	24–60 years	25–55 years	25–57 years	0.99
Weight (kg)	90.4 ± 3.4	94.1 ± 3.2	92.4 ± 3.4	0.73
BMI (kg/m^2^)	30.6 ± 0.7	31.8 ± 0.7	30.9 ± 0.7	0.50

Values reported as mean ± SEM. p-value obtained from one-way ANOVA. BMI, body mass index.

### Diet intervention and ketone supplement

This was a double-blind (both participants and outcome assessors were blinded for the KS portion of the study) controlled feeding study. Study dietitians remained unblinded in order to manage calorie intake of the groups accordingly. All food was prepared in a state-of-the-art metabolic kitchen. All the ingredients were precisely weighed (± 0.1 g), with custom macro- and micronutrients calculated by a team of registered dietitians. Both ketogenic diets were designed based on previous well-formulated standards (Volk et al., 2014; Hyde et al., 2019) and the LFD was developed in accordance with the USDA’s Dietary Guidelines for Americans 2015–2020 (United States Dietics Association, 2015). Menus for each participant were calculated from a base calorie level of 2,000 and scaled to match 75% of estimated energy requirements based on assessment of resting energy expenditure measured by indirect calorimetry (ParvoMedics TrueOne, Salt Lake City, UT, United States, v2400), a standard Harris–Benedict equation for estimating energy expenditure, and the energy cost of their physical activity (Harris and Benedict, 1918).

Protein was set at 1.5 g/kg reference weight for all three diets (Metropolitan, 1983). A portion of the protein for the KD groups was provided as two daily chocolate or vanilla shakes that contained whey protein isolate (∼15 g/serving) along with fat in the form of high oleic sunflower oil and medium chain fatty acids (MCT–caprylic and capric acids, Metagenics, Inc., Aliso Viejo, CA, United States). In addition, subjects in KD groups consumed one serving of 10 g MCT oil with their breakfast and one serving in the afternoon snack.

Both KD groups were provided ∼40 g/day of carbohydrates and the remaining calories were provided as fat, with an emphasis on monounsaturated and saturated fat sources. The LFD provided 25% total fat with <10% saturated fat, at least 32 g fiber/day, ∼100 g of simple carbohydrates (<25 g added sugars) and <30 g added oils/day. *Ad libitum* intake of calorie-free/salt-free products, such as diet soda, non-caloric flavored water, etc., were allowed during the entire intervention. A variety of whole foods were used to develop both the ketogenic and low-fat meals. Average nutrient intakes over the 6 weeks for the three groups were reported previously (Buga et al., 2021).

**FIGURE 1:**
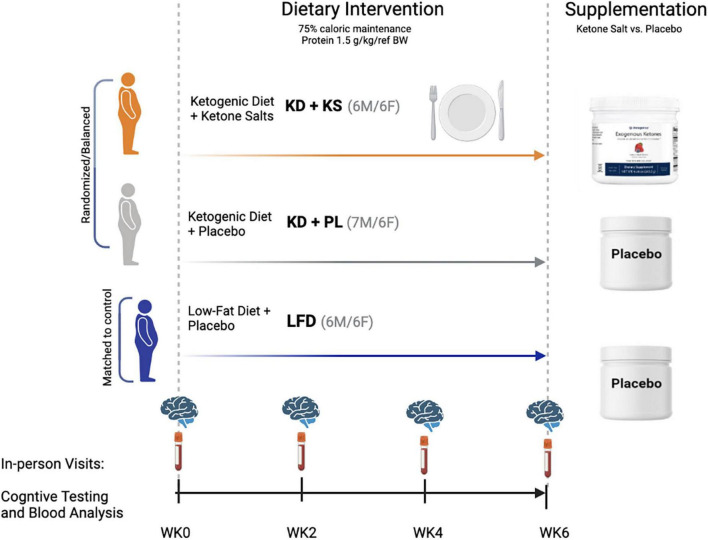
Experimental design and macronutrient breakdown for both ketogenic (KD) and low-fat (LFD) diet groups.

The KD + KS group consumed a ketone supplement consisting of racemic ketone salts (11.8 g BHB with sodium, calcium, and magnesium, Metagenics, Inc., Aliso Viejo, CA, United States) with non-caloric flavoring, shown to acutely increase ketosis in healthy adults (O’Connor et al., 2018). Two doses were taken daily, in the morning and after lunch. Because the ketone supplements contained calories in the form of BHB, the fat content of the KD + KS group’s menu was reduced by ∼120 kcal/day to ensure all 3 diets had similar caloric values. The KD + PL and LFD groups received a calorie-free flavored placebo that contained no BHB or minerals and was identical in appearance and taste to the ketone supplement to maintain the double-blind nature of the study. Experimental design for the study can be found in Figure 1.

### Behavior and cognition

Behavioral surveys and cognitive testing batteries were given biweekly at WK0 (baseline), WK2, WK4, and WK6. Shortened Profile of Mood States (POMS) and Dynamic Visual Analog Mood Scale (D-VAMS) surveys were administered on a computer and used to quantify the following surrogates of mood and behavior: anger, anxiety, depression, fatigue, happiness, restfulness, and vigor (Wyrwich and Yu, 2011; Barrows and Thomas, 2018; de Andrés Terán et al., 2019).

Cognition was measured by Automated Neuropsychological Assessment Metrics (ANAM), a military-grade software that evaluates cognitive response to attention, concentration, reaction time, memory, processing speed, and decision making (Reeves et al., 2007). The test was administered on a computer and the automated protocol allowed for demonstration and practice prior to data collection for each test. The testing battery and descriptions can be found in Table 2.

**TABLE 2:** Automated neuropsychological assessment measures (ANAM) test battery description.

Test	Cognitive domains	Description
**Reaction time**	Reaction time, motor speed, and attention	This test measured simple reaction time by presenting the users with a series of “*” symbols and instructing them to respond as quickly as possible by pressing a button each time the symbol appeared. The main dependent measure is based on the percent error rate.
**Code substitution- (CSD)**	Visual perception, memory, processing speed, and attention	This test asks the user to compare a displayed symbol-digit pair with a set of defined symbol-digit pairs (the key) presented at the top of the screen. The user presses designated buttons to indicate whether the pair in question represents a correct or an incorrect mapping relative to the key. In the Learning phase, the defined pairs are presented on the screen simultaneously with the symbol-digit stimulus in question.
**Procedural reaction time (PRT)**	Processing speed, reaction time, and attention	Subject was presented with a number (2–5) and was asked to press one designated button for a low number (2 or 3) or another for a high number (4 or 5).
**Mathematical processing (MTH)**	Concentration, processing speed, decision making	This task required the participants to perform basic arithmetic operations in the form of “x + y−z = .” The participant indicated whether the solution to the problem was greater than or less than 5.
**Matching to sample (M2S)**	Processing speed and memory	The user viewed a pattern produced by 8 shaded cells in a 4 + 4 sample grid. The sample was then removed, and 2 comparison patterns were displayed side by side. One grid was identical to the sample grid and the other grid differed by one shaded cell. The user was instructed to select the grid that matched the sample.
**Simple reaction time repeated (SR2)**	Reaction time	This is a repeat of the SRT presented earlier in this battery (see above).
**– Delayed code substitution memory**	Learning, attention and delayed visual recognition memory	The user was asked to compare a displayed symbol-digit pair with the set of previously defined and memorized symbol-digit pairs (the key). The user pressed designated buttons to indicate whether the pair in question represented a correct or an incorrect mapping with respect to the memorized key.
**Go/no-go (GNG)**	Response inhibition	The subject was presented with two characters, “x” and “o.” The subject responded as quickly as possible to the “x” by pressing a button each time it appeared. When the “o” appeared, the user was instructed to do nothing.

Based on Levinson et al. (2005) and Reeves et al. (2007).

### Biochemical analysis

#### Beta-hydroxybutyrate and glucose levels

Fasting BHB and glucose concentrations [previously reported in Buga et al. (2021)] were assessed in capillary blood using reagent strips and a monitoring device (Abbot FreeStyle^®^, Columbus, OH, United States). Outside of testing days, participants reported daily fasted ketones every morning during the study *via* image texts sent to research staff. On testing days, fasting venous blood samples were collected *via* venipuncture in the antecubital fossa, performed by a qualified phlebotomist.

#### Adipokine panel

Fasted adipokine biomarkers in human serum (BDNF, IL-1β, IL-6, IL-8, IL-10, Insulin, Leptin, MCP-1, TNF-α, β-NGF) were analyzed using the Meso Scale Discovery’s U-Plex Human Adipokine Panel (Rockford, Maryland). Qualification is based on multi-array technology with a proprietary combination of electrochemiluminescence detection and patterned arrays. The electrochemiluminescence detection used labels that emit light when electrochemically stimulated. Multiple excitation cycles of each label amplify the signal to enhance light levels and improve sensitivity. U-Plex linkers are first coupled to biotinylated capture antibody. This solution is used to coat each 10-spot well in the 96-well plate to appropriately bind the sample. Samples were run in duplicates. The addition of the antibody solution was used to couple the markers to each biomarker spot in the well. The plate was analyzed using the MSD Workbench 4.0 software. Calculations were run using a 4-parameter logistic with 1/y^2^ weighting, and CVs for the standard and samples was 3.6%.

### Statistical analysis

Statistical analyses were performed using GraphPad Prism 5.01 (La Jolla, CA, United States). Two-tail α significance was set at *p* < 0.05. Baseline measures were compared for differences with one-way analysis of variance (ANOVA). Two-way repeated measures (RM) ANOVA was used with diet (KD + KS, KD + PL, and LFD) and time (weeks 0, 2, 4, and 6) as independent variables. Bonferroni’s test was used for all *post-hoc* comparisons. All data are presented as mean ± SEM.

## Results

### Beta-hydroxybutyrate, glucose and insulin levels

**FIGURE 2:**
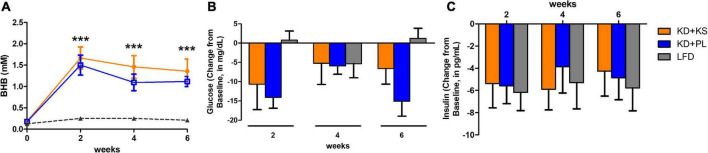
Fasting beta-hydroxybutyrate [BHB, panel **(A)**], glucose **(B)** and insulin **(C)** concentrations collected on testing days over the course of 6 weeks. Values at each week represent mean ± SEM. KD, ketogenic diet; KS, ketone supplement; PL, placebo; LFD, low-fat diet. ***p < 0.001 from baseline (week 0) representing significant differences in both KD groups (KD + KD and KD + PL) compared to LFD group.

Fasted capillary BHB concentrations, which were assessed at least 8 h from the last KS dose, increased progressively in both KD groups throughout the study and remained unchanged in the LFD group (Figure 2A). Two-way RM ANOVA showed main effects of diet (*p* < 0.0001) and time (*p* < 0.0001) and an interaction between diet and time (*p* = 0.001). The ketone supplemented group (KD + KS) showed a trend for higher BHB values (*p* = 0.079), compared to the placebo group (KD + PL).

Glucose levels were reduced over the 6-weeks for both KD groups and, despite fluctuations across time points, were unchanged in the LFD group at the end of the study (Figure 2B). A main effect of time (*p* = 0.001) and interaction between diet and time (*p* = 0.007) were noted, mostly attributed to lower glucose levels in KD + PL when compared to LFD group at weeks 2 and 6 (*p* < 0.01).

All dietary interventions similarly reduced insulin levels across all time points [main effect of time (*p* < 0.0001), Figure 2C].

### Profile of mood states and visual analog mood scales

**FIGURE 3:**
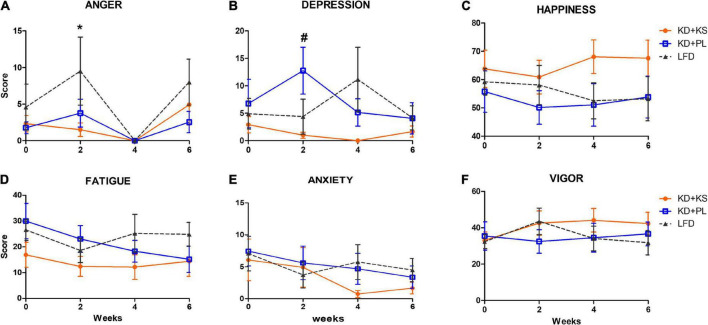
Profile of Moods assessments in all diet groups. **(A)** Increased anger scores were observed in LFD group when compared to KD at week 2. Depression scores **(B)** were higher at week 2 for KD + PL group, compared to KD group supplemented with KS. No statistically significant differences were observed in happiness **(C)**, fatigue **(D)**, anxiety **(E)**, and vigor **(F)** scores. Values represent mean ± SEM. KD, ketogenic diet; KS, ketone supplement; PL, placebo; LFD, low-fat diet. Data included in Supplementary Table 1 but not shown in this figure: Sleepiness, restlessness. *p < 0.05 vs. LFD, ^#^*p* < 0.05 vs. KD + KS.

There were no group differences in POMS and VAMS scores at baseline. Figure 3 summarizes main findings, and complete results are listed in Supplementary Table 1.

Anger scores fluctuated over time with an increase in LFD at week 2 compared to KD + KS [main effects of diet (*p* = 0.04) and time (*p* = 0.015), Figure 3A]. All groups reported absence of anger at week 4, with anger scores increasing at week 6.

A significant interaction between diet and time was noted for depression scores (*p* = 0.04, Figure 3B). *Post-hoc* analysis indicated that the KD + PL group showed higher depression score at week 2 compared to KD + KS (*p* < 0.05). LFD had a non-significant increase in depression scores at week 4. Throughout the study, the KD + KS group demonstrated lower depression scores compared to other groups. Similarly, happiness scores were consistently higher in KD + KS group compared to both KD + PL and LFD groups, albeit this difference did not reach statistical significance (Figure 3C). No main effects of diet, time or interactions were observed for the following outcomes: fatigue (Figure 3D), anxiety (Figure 3E), vigor (Figure 3F), sleepiness (not shown), and restlessness (not shown).

### Automated neuropsychological assessment measures

Due to different baseline scores among groups, results are shown as change from baseline ± SEM. Absolute values and main effects for ANAM results can be found in Table 3 below.

**TABLE 3:** Main effects and interactions for ANAM cognitive battery of tests.

Test	Diet	WK0	WK2	WK 4	WK6	ΔPre–post	Diet	Time	Interaction
RT score	**KD + KS**	249.5 ± 5.6	236.5 ± 5.4	246.6 ± 9.1	244.9 ± 9.2	−5 ± 3	**0.01**	0.096	0.96
	**KD + PL**	275.54 ± 17.2	254 ± 8.1	254.8 ± 9.2	262.8 ± 13.8	−13 ± 6			
	**LFD**	239 ± 9.3	225.4 ± 3.6	229.9 ± 8.1	237.5 ± 5.9	−2 ± 3			
Code subst score	**KD + KS**	1,109.5 ± 69.4	1,053.9 ± 106.4	1,010 ± 84	1,088.4 ± 121.7	−21 ± 40	**0.04**	**<001**	0.15
	**KD + PL**	1,270.2 ± 106.2	1,200.9 ± 131.9	1,089.3 ± 112.2	1,021.2 ± 112.5	−249 ± 45			
	**LFD**	992.2 ± 46.6	802.9 ± 31.0	815.8 ± 34.4	783 ± 25.9	−209 ± 15			
PRT score	**KD + KS**	556.7 ± 19.3	554.3 ± 23.4	539.6 ± 22.1	505.5 ± 26.2	−51 ± 9	**0.02**	**0.115**	0.89
	**KD + PL**	646.2 ± 55.8	611.5 ± 30.5	597.8 ± 38	589.6 ± 32.5	−57 ± 19			
	**LFD**	521.6 ± 11.4	524.3 ± 20.2	514.8 ± 16.9	514.4 ± 18.6	−7 ± 6			
MP score	**KD + KS**	2,664.3 ± 241.7	2,546.5 ± 185.1	2,413.5 ± 216.1	2,447.4 ± 180.4	−217 ± 87	**0.05**	**<0.001**	**0.02**
	**KD + PL**	3,293.1 ± 386.1	3,033.2 ± 273.6	2,531.5 ± 276.2	2,226.2 ± 237.6	−1,067 ± 131			
	**LFD**	2,459.2 ± 166.6	1,950.6 ± 131.6	1,880.8 121.2	1,941.1 ± 119.5	−518 ± 59			
MTS score	**KD + KS**	1,784.1 ± 143.3	1,524.5 ± 179.3	1,514 ± 93.8	1,528.9 ± 132.2	−255 ± 56	0.16	**0.012**	0.49
	**KD + PL**	1,632.1 ± 106.7	1,510.6 ± 134.1	1,646 ± 217.2	1,366.6 ± 159.5	−266 ± 55			
	**LFD**	1,382 ± 77.2	1,286.2 ± 77.3	1,280.4 ± 57.3	1,250.1 ± 52.9	−132 ± 27			
DCS score	**KD + KS**	1,186 ± 73.1	1,091.5 ± 80.4	1,145.7 ± 91.3	1,081.4 ± 88.5	−105 ± 33	0.12	**<0.001**	**0.02**
	**KD + PL**	1,416.3 ± 166.9	1,108.5 ± 144.7	1,081.5 ± 123.7	1,019.1 ± 113.2	−397 ± 58			
	**LFD**	940 ± 34.8	929.8 ± 71.8	893.8 ± 65.5	864.8 ± 46.6	−75 ± 17			
SRT score	**KD + KS**	243.1 ± 7.7	254.0 ± 9.1	255.6 ± 11.4	268.9 ± 21.9	26 ± 7	0.16	**0.006**	0.36
	**KD + PL**	252.2 ± 6.7	302.7 ± 21.9	284.7 ± 22.0	274.1 ± 17.6	22 ± 5			
	**LFD**	236.5 ± 3.3	278.3 ± 11.1	249.3 ± 13.4	243.9 ± 7.6	7 ± 2			
GNG score	KD + KS	311.3 ± 6.4	274.1 ± 25.3	306.5 ± 8.6	305.6 ± 8.6	−6 ± 3	0.27	**<0.001**	0.95
	KD + PL	319.7 ± 10.5	292.1 ± 131.5	325.8 ± 14.5	330.4 ± 17.3	11 ± 6			
	LFD	302.8 ± 6.3	252.4 ± 12.1	306.8 ± 10.8	294.6 ± 8.2	−8 ± 3			

All data presented as mean ± SEM. Significant main effects from Two-way RM ANOVA and interactions (p < 0.05) are denoted in bold.

**FIGURE 4:**
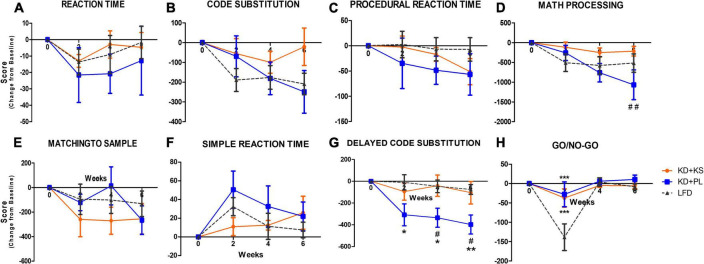
Automated neuropsychological assessment measures (ANAM) results for different cognitive domains in all diet groups. Change from baseline values are used to correct for different baseline values within groups. **(A)** Reaction time, **(B)** code substitution, **(C)** procedural reaction time, **(D)** math processing, **(E)** matching to sample, **(F)** simple reaction time, **(G)** delayed code substitution, and **(H)** Go/No-Go. Values represent mean ± SEM. KD, ketogenic diet; KS, ketone supplement; PL, placebo; LFD, low-fat diet. Data included in Table 3 but not shown in this figure: code substitution (% Correct), Procedural reaction time (% Correct), math processing (% Correct), matching to sample (% Correct), delayed code substitution (% Correct), and Go/No-Go (% Correct, Hits, Omissions). *p < 0.05 vs. LFD, **p < 0.001 vs. LFD, ***p < 0.001 vs. LFD, ^#^*p* < 0.05 vs. KD + KS, ^##^*p* < 0.01 vs. KD + PL.

No main group differences were seen for reaction time scores but there was a trend for a main time effect (*p* = 0.09, Figure 4A), indicating a modest decrease in reaction time across all three groups, reflecting positive response during the intervention that was comparable across all groups. Code substitution scores improved over time (time effect, *p* < 0.001, Figure 4B) but no significant group differences were detected.

No diet or time effects were observed for procedural reaction time scores (Figure 4C). Main effect of interaction between diet and time were revealed for math processing (*p* < 0.0001, Figure 4D) where *post-hoc* analysis showed that participants in KD + KS group had significant higher scores than subjects in KD + PL group at week 6 (*p* < 0.01). Both matching to sample and simple reaction time scores were also significantly impacted by time (*p* = 0.01, Figures 4E,F, respectively) but no group differences were evident. Delayed substitution scores were affected by both diet (*p* = 0.01), time (*p* < 0.0001) and interaction between diet and time (*p* = 0.02, Figure 4G), as evidenced by a significant decline for KD + PL participants, reaching statistical significance compared to both KD + KS [weeks 4 and 6 (*p* < 0.05)] and LFD [weeks 2, 4 (*p* < 0.05) and 6 (*p* < 0.01)]. Moreover, main effects of diet (*p* = 0.05), time (*p* < 0.0001) and interaction between diet and time (*p* = 0.002) were observed for go/no-go scores (Figure 4H). KD + KS group showed significantly higher go/no-go scores at week 2 when compared to LFD group, while not statistically different from KD + PL.

Supplementary Table 1 includes all results and two-way RM ANOVA analyses, including raw score values and the following additional tests not included in Figure 4: code substitution (% correct), procedural reaction time (% correct), math processing (% correct), matching to sample (% correct), delayed code substitution (% correct), go/no-go (% correct, hits, omissions).

### Adipokine responses

**FIGURE 5:**
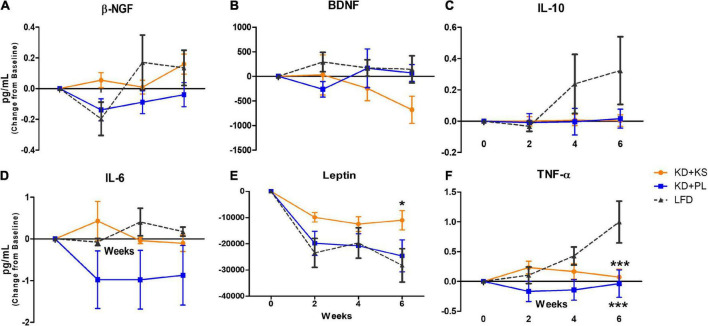
Quantification of adipokine biomarkers in human plasma in all diet groups. Results are presented (in pg/mL) as change (± SEM) from baseline values. **(A)** β-NGF, **(B)** BDNF, **(C)** IL-10, **(D)** IL-6, **(E)** Leptin, and **(F)** TNF-α. KD, ketogenic diet; KS, ketone supplement; PL, placebo; LFD, low-fat diet. Data included in Supplementary Table 1 but not shown in this figure: IL-6, IL-8, IL-1β, and MCP-1. *p < 0.05 vs. LFD, ***p < 0.001 vs. LFD.

Due to heterogeneity in baseline values across all groups, the adipokine results are presented as change from baseline (Figure 5). Absolute values and statistical results are listed in Supplementary Table 1. Main effects of time were noted for β-NGF (*p* < 0.01, Figure 5A), and leptin (*p* < 0.0001, Figure 5E). Moreover, significant interactions between diet and time were observed for β-NGF (*p* = 0.028, with no *post-hoc* differences) and leptin (*p* = 0.05), due to subjects in the KD + KS group showing higher leptin levels than subjects in the LFD group at week 6 (*p* < 0.05).

No differences between groups or over time were detected for BDNF (Figure 5B), IL-10 (Figure 5C), IL-6 (Figure 5D), IL-1β (not shown), IL-8 (not shown), and MCP-1 (not shown).

TNF-α levels (Figure 5F) were impacted by diet (*p* = 0.02) and time (*p* = 0.01) and an interaction between diet and time was observed (*p* = 0.001). While TNF-α levels showed non-significant fluctuations in both KD groups, it steadily increased for LFD participants, reaching statistical significance at week 6 (*p* < 0.001).

## Discussion

A primary finding in this exploratory study was that there was no indication of a consistent decline in various measures of mood or cognitive function during a short-term 6-week hypocaloric KD, despite being relatively low in sodium relative to a low-fat diet. The addition of a KS to the KD modestly elevated circulating BHB beyond and provided considerably more sodium, which may have contributed to decreased depression scores and higher happiness scores in this group. Specific changes were seen at WK2 behavioral markers showing that KD + PL has significantly higher depression scores and LFD reported significantly higher anger scores, compared to KD + KS group. Cognitive performance was generally stable during weight loss across all diets.

Both KD groups reached nutritional ketosis by the first week with BHB levels above 1.0 mM, as reported in Buga et al. (2021) and the ketone salt supplemented group displayed a non-significant trend for slightly higher levels of BHB. It has previously been reported that the KS used in this investigation acutely and transiently increased BHB to approximately 1.0 mM 1-h after intake, while fasted (O’Connor et al., 2018). Thus, the levels of ketones determined from overnight fasted samples likely underestimate the transient increase in the overall exposure to higher ketosis during the day.

Glucose-lowering is a well-established benefit of ketogenic diets (Yuan et al., 2020; Hyde et al., 2021), and this effect was not augmented by the addition of ketone salts. On the other hand, participants that followed the LFD showed glucose fluctuations with decreased glucose levels at week 4 that returned to baseline levels at week 6. Higher glucose levels, even within upper range of clinically considered normal values, has been associated with higher risk for dementia onset (Crane et al., 2013). Therefore, lowering and stabilization of fasting glucose levels is a clinically advantageous benefit of KDs that intersects both metabolic and cognitive health. Our findings support the notion that KDs are likely a promising preventative therapeutic approach for individuals at higher risk of developing cognitive impairments *via* modulation of fasting glucose levels (Huttenlocher, 1976; VanItallie et al., 2005; Crane et al., 2013; Taylor et al., 2018).

Insulin dysregulation plays a key role in cognitive decline in patients with AD (Cholerton et al., 2013; Kellar and Craft, 2020) and psychiatric disorders (Penninx and Lange, 2018) in such a way that peripheral insulin resistance decreases the ability of the hormone to cross the blood brain barrier (Schiöth et al., 2012). We suspected that the hypocaloric nature of this study would reduce insulin levels for all groups (Clamp et al., 2017), but that KD interventions would further augment the effect. Our results showed that all dietary interventions tested successfully and similarly decreased insulin levels, an effect that was likely attributed to weight-loss and independent of KS inclusion. Likewise, a recent study with type-2 diabetic population following a low-carbohydrate lifestyle reported decreased subclinical depressive symptoms as early as 10 weeks with reductions maintained up to 2 years (Adams et al., 2022). While this is a research area of growing interest, limited clinical studies so far have been conducted with this patient population. Emerging areas with clinical interest and mechanistic plausibility for KD as a therapeutic intervention include anxiety, depression, bipolar disorder, schizophrenia, autism spectrum disorder and attention hyperactivity disorder [reviewed in Bostock et al. (2017) and Kovács et al. (2019)].

We hypothesized that augmentation of ketosis by twice daily ingestion of a KS would facilitate the transition during the keto-adaptation weight loss phase, similarly to what was reported using an MCT-supplemented KD (D C. Harvey et al., 2018). Commonly reported “keto-flu” symptoms include headaches, fatigue, brain fog, decreased energy among others, peaking in the first few days and lasting up to 4 weeks (Bostock et al., 2017). A key physiological event associated with keto flu symptoms is the initial mineral imbalance that occurs during the keto adaptation phase, which is driven by the natriuretic and diuretic effects of very low-carbohydrate ketogenic diets. While KD + PL group met dietary guideline RDA for sodium, previous literature suggests that KDs may require a higher intake of sodium due the natriuretic effects of the diet (O’Donnell et al., 2014; Volek et al., 2021). Hyponatremia has a known association with depressive mood in clinical populations (Fan et al., 2020; Suárez et al., 2020). Indeed, our findings showed that supplementation with KS containing a proprietary combination of minerals elicited subjective mood amelioration, reflected here as lower depression and higher happiness scores (Figure 3), particularly when compared to subjects following a KD without KS. Moreover, subjects following LFD reported higher anger scores at week 2 compared to KD + KS group but no differences between KD groups were seen.

Carbohydrate-restricted diets have been previously associated with fatigue and reduced vigor, particularly in response to exercise, compared to non-ketogenic low-carbohydrate diets. A 2006 study reported unfavorable mood profile, fatigue, and reduced vigor in KD group (Johnston et al., 2006). There was no mention of sodium content of the KD, reported potassium levels were deficient, and the KD failed to achieve the lower threshold of nutritional ketosis (Johnston et al., 2006). In the current study, we did not observe significant differences in either fatigue or vigor across all three dietary interventions, possibly due to the well-balanced nature of the KD.

The ANAM battery of cognitive tests was chosen based on previous evidence that it is a valid and reliable tool to assess cognitive function in military adult population (Levinson et al., 2005; Reeves et al., 2007). Our group recently established that high-intensity resistance exercise may elicit domain-specific influences on cognitive function utilizing the ANAM core test battery (Anders et al., 2021). Performance in several tests were improved throughout the 6-week study, showing familiarization, and learning of specific tasks. For instance, reaction time decreased while its scores improved, and code substitution and matching to sample scores improved over time, regardless of dietary group. Math processing scores were negatively impacted in KD + PL group at the end of the study whereas both KD + KS and LFD groups showed consistent scores in this task throughout the 6-week study.

Our findings showed that both LFD and KD + KS resulted in consistent performance in tasks that required both attention and visual recognition memory. At week 2, Participants following the LFD showed a significant detriment in the go/no-go task which entails response inhibition, an effect that was not observed in either KD dietary intervention group. Most evidence to date supporting the use of ketogenic approaches as a therapeutic option to attenuate cognitive decline has been derived from studies performed in populations with cognitive impairments at baseline or in the presence of another neurological disorder. Subjects in our study were between 21 and 65 years and cognitively healthy at baseline, which likely explains the slight changes observed in cognitive outcomes and lack of additional benefits of twice-daily KS intake.

The literature on the effects of ketogenic interventions on BDNF have been inconsistent and contradictory, particularly when it comes to comparisons between peripheral or cerebral BDNF levels. Walsh et al. found that after administration of a ketone ester (KE) there was no change in BDNF concentrations in healthy populations but later saw either sustained or increased BDNF following both KE ingestion and an Oral Glucose Tolerance Test in normal and overweight participants (Walsh et al., 2020, 2021). Two studies showed increased BDNF concentrations after a >8-week KD intervention in healthy weight adults (Mohorko et al., 2019; Paoli et al., 2021). Elevated NGF-β and BDNF concentrations are often discovered in obese populations during metabolic panel screening (Levinger et al., 2008; Atanassova et al., 2014; Lee et al., 2016). Evidence attributes this effect to NGF-β and BDNF overexpression to confer neuroprotective for the central nervous system, thereby mitigating cognitive decline. Weight loss has been linked to a decrease in BDNF levels, returning them to healthy range, which corresponds to the changes that we detected in KD + KS (Levinger et al., 2008; Lee et al., 2016). NGF-β concentrations, on the other hand, were differentially affected by dietary interventions, with fluctuations throughout the experimental procedure but not statistically significant at any of the testing days. Collectively, neuropeptide changes in our intervention were likely attributed to weight-loss rather than diet composition *per se*. Future studies should consider pre-screening for NGF-b and BDNF to quantify if baseline neuropeptide levels are directly proportional to the magnitude change during an intervention (i.e., higher NGF-b and BDNF concentrations are expected to decrease more over time vs. normal physiological concentrations).

Leptin is one of the most studied adipose-derived hormones and considered an important link between metabolic health, cognitive function and inflammation (Matarese et al., 2005; Warren et al., 2012; Monda et al., 2020). Leptin It is also found to be over-expressed in patients with metabolic syndrome synergistically with overexpression in BDNF and NGF- β (Levinger et al., 2008; Atanassova et al., 2014; Lee et al., 2016). As expected with weight loss, all groups showed a drop in serum leptin throughout the study, however, the magnitude of decline was attenuated in the KS group, particularly when compared to LFD leptin levels at week 6. Considering that leptin levels are directly associated with BMI and fat mass and given that no overall metabolic differences were present between the two KD groups regarding weight loss (Buga et al., 2021) and changes in liver fat content (Crabtree et al., 2021), we speculate whether the regulatory effect of the KD with adequate mineral intake from the supplement may be the reason for the significant difference in leptin levels between groups. Additional experiments aiming to better understand adipose-tissue responses to chronic supplementation with ketone salts will help elucidate the neuroregulatory and inflammatory mechanisms involved in this pathway.

Of interest, the pro-inflammatory cytokine TNF-α steadily increased in the LFD group throughout the study, reaching statistical significance at week 6, compared to both KD groups; a similar non-significant trend was observed for the anti-inflammatory IL-10 cytokine. High systemic TNF-α levels correspond with rapid cognitive decline over 6 months in AD patients, thus dietary modulation of this cytokine should be further investigated (Hennessy et al., 2017). Chronic inflammation in obese populations is a key contributor to elevated TNF -α levels. Weight loss is a typical strategy used to reverse chronic inflammatory responses (Jung and Choi, 2014), but weight loss itself may not be enough to decrease levels in those with comorbidities (Di Francia et al., 1994), therefore the anti-inflammatory nature of the KD may benefit this patient population (Newman and Verdin, 2014; Dupuis et al., 2015).

A considerable strength of our study was that the controlled-feeding design ensured that participants adhered to the assigned dietary approach while daily ketone assessments could be construed as an objective biomarker to track compliance. Moreover, dietary plans were carefully designed by a team of registered dieticians and palatability was a contributing factor in the high adherence observed. One of the common shortcomings of dietary studies is the lack of consistency of meal and ingredient recommendations and our approach successfully addressed this challenge.

The 6-week timeframe was chosen due to successful body weight and metabolic changes observed in previous KD studies and the dietary interventions tested indeed resulted in improved metabolic and body composition findings (Volek et al., 2002; Buga et al., 2021; Crabtree et al., 2021). However, 6 weeks may not have been sufficient to elicit pronounced cognitive changes, particularly in a cognitively healthy adult population. Additionally, it is unclear what the long-term results would suggest if weight-maintenance was attained, given that all the primary outcomes reported in this publication have been collected during consistent, bi-weekly weight-loss. Despite slight amelioration of mood outcomes, KS did not significantly or consistently impact cognitive function, likely due to its modest and transient increase in circulating BHB levels. Further research is needed with the addition of a KD with sodium matched levels of the KD + KS group, without the addition of ketones. This may prevent outcomes associated specifically with higher ketone levels as sodium levels were also increased.

The cognitive battery in this study may have limitations. Firstly, the only available normative data for the ANAM is from a military sample set. Although, we have previously used ANAM in general population research, normative scores were not calculated for this sample. Lastly, even considering the test–retest reliability for ANAM is positive, taking the test 4 times within a 6 week period may have introduced varying practice effects, which could explain some level of variation in individual performance outcomes over time (Vincent et al., 2018).

## Conclusion

In summary, results of our exploratory investigation suggest that the inclusion of a KS to a well-formulated hypocaloric KD can attenuate detriments in mood compared to KD + PL and LFD interventions, with no distinct differences in cognitive function. It is possible that the KS provided adequate mineral supplementation that the KD + PL group needed and higher BHB levels than the LFD group, resulting in overall more positive scores in the KD + KS group during weight loss. Adipokines associated with both metabolic and cognitive issues responded differently in the KD + KS and LFD groups and thus require further investigation.

## Data availability statement

The original contributions presented in this study are included in the article/Supplementary material, further inquiries can be directed to the corresponding author.

## Ethics statement

The studies involving human participants were reviewed and approved by The Ohio State University Biomedical Sciences IRB. The patients/participants provided their written informed consent to participate in this study.

## Author contributions

JV, MB, MK, and AO’C: conception of study design. MK, AB, CC, and TS: conducting human subject experiments. MB, MK, and JV: analyzing and interpreting data and writing initial manuscript drafts. All authors participated in critical editing and approved the final version of the manuscript.
